# The Pattern of Adverse Drug Reaction Reporting at a Regional Pharmacovigilance Center in North India: A Retrospective Observational Study

**DOI:** 10.7759/cureus.91812

**Published:** 2025-09-08

**Authors:** Pankaj K Gupta, Jasleen Kaur, Gayatri Devi, Shilpa Chaudhary, Jyoti Maria

**Affiliations:** 1 Pharmacology, Employees’ State Insurance Corporation Medical College and Hospital, Faridabad, Faridabad, IND; 2 Pharmacology/Pharmacovigilance, Employees’ State Insurance Corporation Medical College and Hospital, Faridabad, Faridabad, IND; 3 Pharmaceutical Sciences, Delhi Pharmaceutical Sciences and Research University, New Delhi, IND

**Keywords:** adverse drug reactions, causality assessment, patient safety, pharmacovigilance, severity of adr

## Abstract

Background

Adverse drug reactions (ADRs) are one of the common causes of morbidity and mortality and contribute significantly to the healthcare burden. However, they are largely preventable. Pharmacovigilance can help alert healthcare providers about possible ADRs. It can also help protect the patients from the probable side effects of medications in the future.

Methodology

This retrospective study included ADRs reported in ESIC Medical College & Hospital, Faridabad, an ADR monitoring center under the National Co-ordination Centre-Pharmacovigilance Programme of India and the Indian Pharmacopoeia Commission, Ghaziabad. The assessment was performed to analyze the pattern of ADRs reported from January 2022 to December 2022. Reports were scrutinized based on patient demographics, drug characteristics, type of ADRs, outcomes, causality, severity, and seriousness. The data were collected and entered into the standard ADR reporting form. Each reported adverse event was assessed individually. Causality was determined using the World Health Organization-Uppsala Monitoring Centre (WHO-UMC) causality assessment scale.

Results

A total of 282 reported/notified suspected ADRs from different departments were analyzed and reported over 12 months. Almost 78% (n = 156) of ADRs were seen in the adult age group (21-60 years). Antibiotics were the most frequently implicated drug class, presenting 36% (n = 72) of ADRs. Causality assessment, vital for linking ADRs to drugs, was performed using the WHO-UMC scale. Overall, 91% (n = 182) of ADRs were classified as “probable” and 8% (n = 16) as “possible,” with no cases categorized as “certain” or “unlikely.”

Conclusions

The study provides important insights into serious ADRs reported in 2022 at a tertiary care center in India. Adults were predominantly affected, with antibiotics, especially cephalosporins, being common culprits. Most ADRs were recognized and labeled, confirming known drug-event relationships, though a few potentially novel or rare ADRs were noted.

## Introduction

An adverse drug reaction (ADR) is an unintended and harmful response to a medication that occurs at doses normally used in humans. These reactions can vary in severity from mild symptoms to life-threatening conditions. Multiple factors influence the occurrence of ADRs, such as individual differences in drug metabolism, drug-drug or drug-substance interactions, allergies, dosing inaccuracies, and pre-existing health conditions. Common manifestations include nausea, dizziness, dermatological reactions, gastrointestinal issues, and allergic responses. Severe ADRs may lead to hospitalization, organ damage, or death.

The World Health Organization (WHO) defines pharmacovigilance as the science and activities involved in detecting, assessing, understanding, and preventing adverse effects or other drug-related problems, particularly concerning both short- and long-term effects of medicines [1]. Continuous monitoring of ADRs is critical for safeguarding patient health and maintaining trust in treatment protocols. In India, the Pharmacovigilance Programme of India (PvPI), established in July 2010, functions as the national framework dedicated to monitoring drug safety and promoting the rational use of medications [2].

ADRs are reported by healthcare professionals, patients, and caregivers. These reports are analyzed to detect potential safety concerns, and cases that meet the PvPI criteria are submitted to the national database [3]. The PvPI’s objective is to improve patient safety by systematically collecting, evaluating, and acting on ADR data to minimize drug-related risks [4].

This study aimed to assess the patterns of ADR reporting, patient age distribution, commonly implicated drug classes and specific medications, frequent symptoms (classified by MedDRA preferred terms), affected organ systems, severity of ADRs, and clinical outcomes. The data were sourced from the regional pharmacovigilance center at ESIC Medical College and Hospital, Faridabad, India.

## Materials and methods

A retrospective, observational, cross-sectional study was conducted at the Regional Pharmacovigilance Centre of ESIC Medical College, Faridabad, Haryana, India. ADR reports submitted between January 1, 2022, and December 31, 2022, were screened based on predefined inclusion and exclusion criteria. Institutional Ethics Committee approval was obtained before initiating the study (approval number: 134X/11/13/2023-IEC/DHR/113).

ADR reports originated from outpatient departments as well as inpatient wards, including cardiology, dermatology, gynecology, hematology, general medicine, ophthalmology, pediatrics, psychiatry, tuberculosis and chest, and neurology departments.

Data were collected in the suspected ADR reporting form (version 1.3) and included patient initials, age, gender, department reporting the ADR, description and duration of the reaction, suspected drug(s) (brand and generic names), and patient outcomes. Causality assessment of each ADR was performed using the World Health Organization-Uppsala Monitoring Centre (WHO-UMC) criteria [5].

Inclusion criteria

All adverse events that met the reporting standards of the PvPI were included [3]. Each report required patient details (age and gender) and reporter details (name or address), identification of at least one suspected drug (with brand and generic names if available), and a comprehensive description of the ADR. Patients of all age groups were eligible, provided relevant medical records or prescriptions were available during the study period.

Exclusion criteria

We did not include any adverse events related to medical devices and medication errors. Adverse events that did not fulfil the minimum reporting requirements as outlined by PvPI [3] were also excluded.

## Results

A total of 200 spontaneous ADR reports were received at the regional pharmacovigilance centre, ESIC Medical College and Hospital, Faridabad. These were analyzed and reported to PvPI during the study period of 12 months. These reports were scrutinized.

Table 1 depicts sources of ADR reports, in which out of 200 reports, 99 were from other peripheral hospitals, one was from a patient/consumer reported directly to the regional pharmacovigilance center, and 100 were from ESIC Medical College and Hospital, Faridabad.

**Table 1:** Sources of spontaneous ADR reports. ADR = adverse drug reaction

Source of spontaneous reports	Number of ADRs reported (N = 200)
Other peripheral hospitals, Faridabad	99
Patient/Consumer	1
ESIC Medical College and Hospital, Faridabad	100

Table 2 depicts that the majority of adverse events were reported in the age group of 31-40 years (n = 51), followed by 21-30 years (n = 38), 41-50 years (n = 35), 51-60 years (n = 32), 11-20 years (n = 17), 61-70 years (n = 14), 1-10 years (n = 6), 71-80 years (n = 4), 81-90 years (n = 1), and not known (n = 2).

**Table 2:** Age distribution of patients experiencing AEs. AE = adverse event

Age distribution of patients experiencing AEs	Number of AEs reported (N = 200)
1–10 years	6
11–20 years	17
21–30 years	38
31–40 years	51
41–50 years	35
51–60 years	32
61–70 years	14
71–80 years	4
81–90 years	1
Not known	2

**Figure 1:**
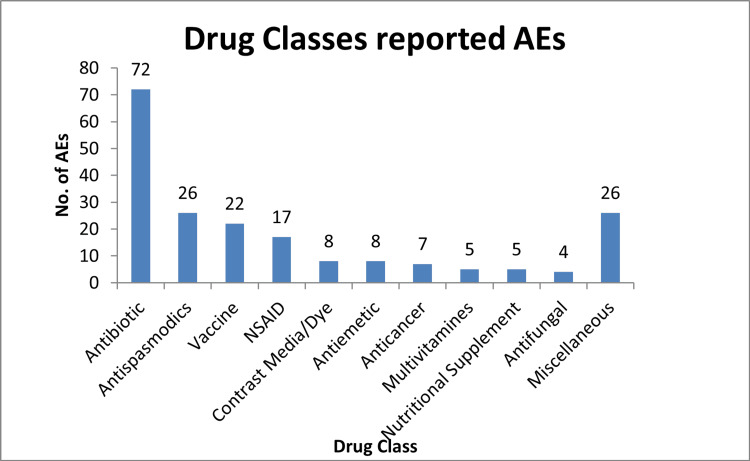
Distribution of AEs by drug class. AE = adverse event; NSAID = non-steroidal anti-inflammatory drug

Figure 1 illustrates that the highest ADRs, i.e., 36% (n = 72), were reported with antibiotics, followed by antispasmodic drugs at 13% (n = 26), vaccines at 11% (n = 22), and non-steroidal anti-inflammatory drugs at 8.5% (n = 17).

**Figure 2:**
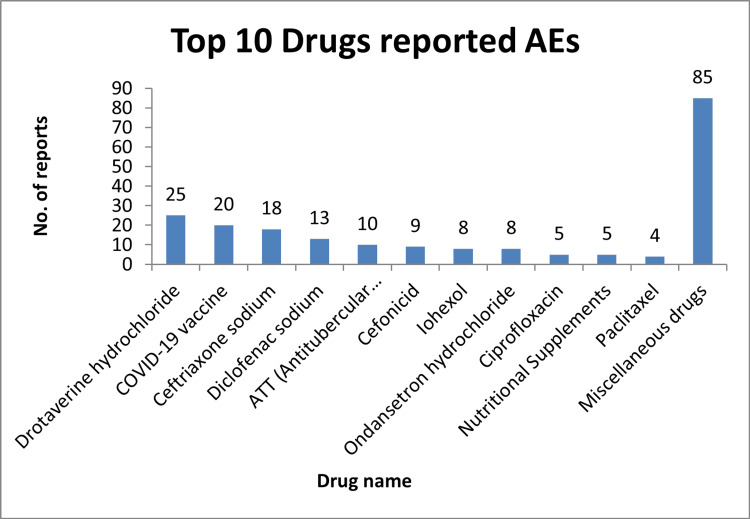
AEs by specific drugs. AE = adverse event

Figure 2 illustrates that the highest adverse events were reported from Drotaverine (n = 25), followed by COVID-19 vaccines (n = 20), ceftriaxone (n = 18), diclofenac sodium (n = 13), anti-tubercular drugs (n = 10), cefonicid (n = 9), iohexol (n = 8), ondansetron (n = 8), ciprofloxacin (n = 5), and the least from paclitaxel (n = 4).

**Figure 3:**
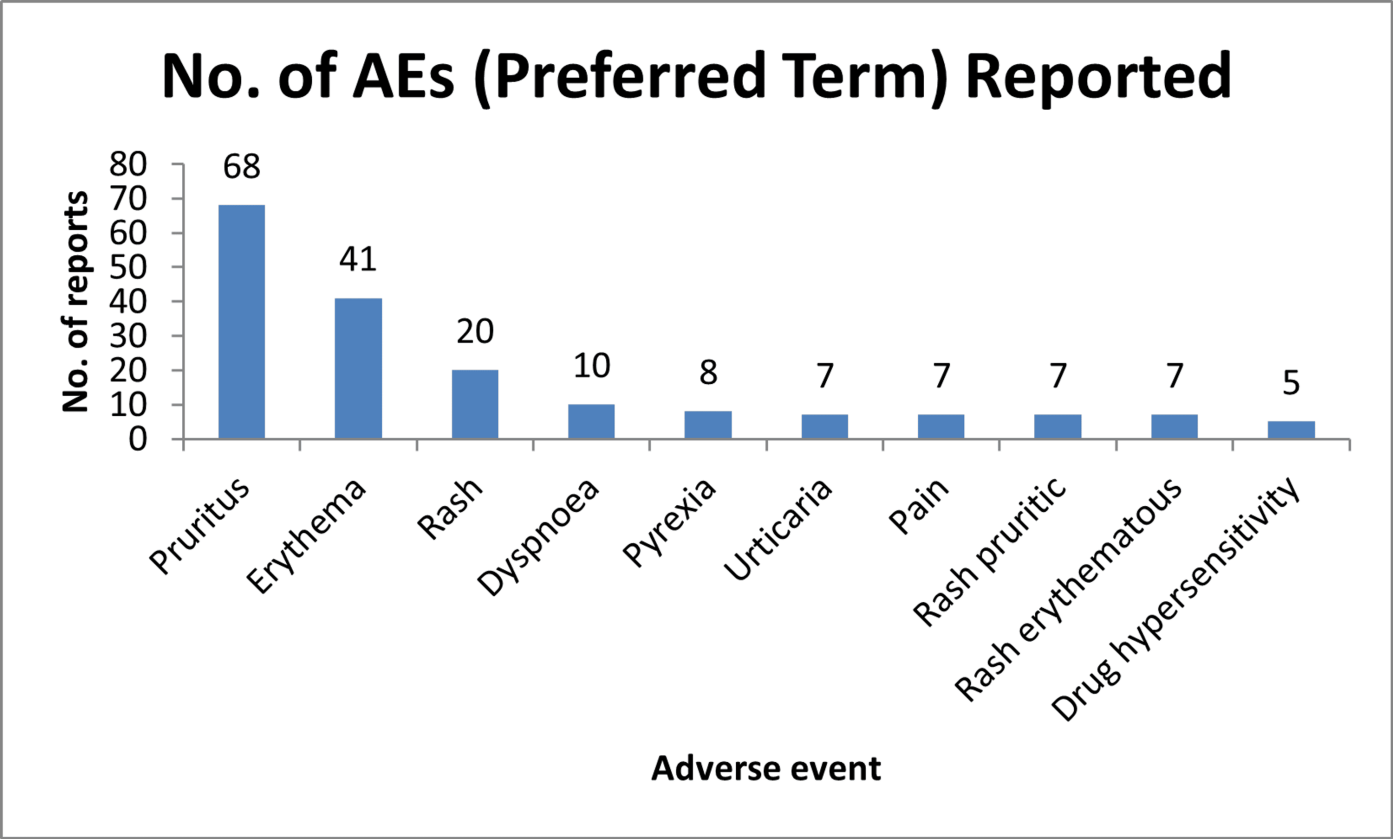
Most commonly reported preferred terms for AEs. AE = adverse event

Figure 3 illustrates that the maximum preferred adverse event terms reported are pruritus (n = 68), erythema (n = 41), rash (n = 20), dyspnoea (n = 10), pyrexia (n = 8), urticaria, pain, rash pruritic, rash erythematous (n = 7), and drug hypersensitivity (n = 5).

Table 3 shows that the maximum ADRs were reported in patients with skin and subcutaneous tissue disorders (n = 163), followed by general disorders and administration sites (n = 27), respiratory system (n = 19), nervous system (n = 14), immune system (n = 12), gastrointestinal system (n = 11), psychiatric disorders (n = 8), musculoskeletal and connective tissue disorders (n = 7), cardiac disorders (n = 5), and injury, poisoning, and procedural complications (n = 3).

**Table 3:** Systemic distribution of ADRs. ADR = adverse drug reaction

Systems	Number of ADRs (n = 282)
Skin and subcutaneous tissue disorders	163
General disorders and administration site conditions	27
Respiratory, thoracic, and mediastinal disorders	19
Nervous system disorders	14
Immune system disorders	12
Gastrointestinal disorders	11
Psychiatric disorders	8
Musculoskeletal and connective tissue disorders	7
Cardiac disorders	5
Injury, poisoning, and procedural complications	3
Miscellaneous	13

Among all ADR reports (N = 200) submitted, there were a total of 282 ADRs reported and analyzed. Of these, 28 drug reactions were serious, and 254 cases were found to be non-serious (Table 4), as per the seriousness criteria such as death, life-threatening, congenital anomaly, hospitalization/prolonged, disability, or other medically important. In our study, the reason behind serious ADRs was other medically important conditions (n = 21) and prolonged hospitalization (n = 7).

**Table 4:** Severity of ADRs. ADR = adverse drug reaction

Seriousness	Number of ADRs reported (n = 282)
Serious	28
Non-serious	254

**Figure 4:**
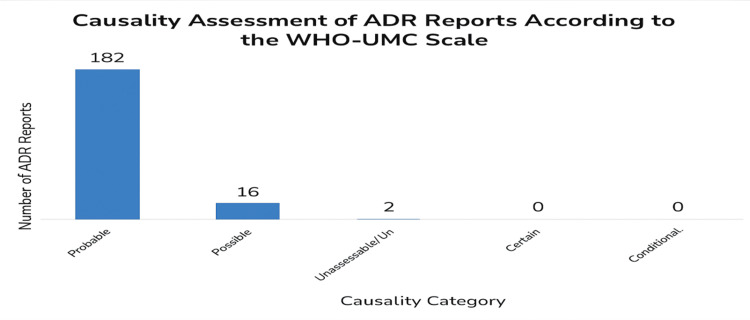
Causality assessment of adverse events (WHO-UMC scale). ADR = adverse drug reaction; WHO-UMC = World Health Organization-Uppsala Monitoring Centre

Figure 4 illustrates the causality assessment where 182 cases were found to be “probable,” 16 cases were “possible,” two cases were “unassessable/unclassifiable,” and no report was considered “certain,” “unlikely,” or “conditional.” All adverse event cases were managed in the respective department from where they were reported, as per standard treatment guidelines.

## Discussion

PvPI operates as India’s national pharmacovigilance initiative, collecting ADR reports from healthcare providers and the public nationwide. It analyzes these reports and communicates relevant safety information to regulatory authorities for appropriate action. Additionally, PvPI disseminates risk information to healthcare professionals and the public to enhance drug safety awareness. Healthcare workers play an essential role in supporting PvPI’s efforts by promoting the safe use of medicines.

This study reviewed ADR reports (N = 200) submitted from January to December 2022 at the ADR Monitoring Centre, ESIC Medical College and Hospital, Faridabad. Age was a significant factor influencing ADR occurrence. Children and elderly patients are particularly vulnerable due to altered drug metabolism and pharmacokinetics, necessitating careful monitoring to reduce ADR risks. Adults aged 21-60 years comprised the majority of ADR (78%, n = 156) cases in this study, consistent with findings reported by Kaur et al. [6] and Meda et al. [7], who reported adult ADR incidences of 75.8% and 71.26%, respectively. On the contrary, Jayanthi et al. [8] reported a higher frequency of ADRs in the elderly, possibly due to demographic differences in study populations.

Antibiotics were the most frequently implicated drug class, accounting for 36% (n = 72) of ADRs, likely reflecting their extensive use. This aligns with Zaman et al. [9] and Ingale et al. [10], who found antibiotic-related ADR rates of 21% and 36.73%, respectively.

The Department of Dermatology contributed about 57.80% (n = 163) of ADR reports, followed by 9.57% (n = 27) of general medicine disorders. The least were reported with cardiac disorders, i.e., 1.77% (n = 5). This contrasts with studies by Nirumalla et al. [11] and Meda et al. [7], which found general medicine as the primary source of ADRs (55% and 56.6%, respectively). Sangeetha et al. [12] similarly reported general medicine as the leading contributor (37.5%).

Causality assessment, vital for linking ADRs to drugs, was performed using the WHO-UMC scale [5]. Most ADRs were classified as “probable” (91%, n = 182), followed by “possible” (8%, n = 16), with no cases categorized as “certain” or “unlikely.” These findings agree with previous studies, though some report higher “possible” proportions. A study from Chennai by Meda et al. found 71% of “probable,” 22% of “possible,” and 4% of “certain” ADRs [13]. The predominance of probable cases likely reflects prompt drug withdrawal and the ethical impracticality of rechallenging patients. These results emphasize the importance of robust pharmacovigilance systems.

Skin and subcutaneous tissue disorders were the most frequently reported ADRs [4,12,14-16], consistent with larger studies [13]. Common symptoms included pruritus, rash, erythema, and dyspnea, which typically resolved fully regardless of severity.

India faces challenges in ADR reporting due to underreporting influenced by fear, lack of awareness, delayed identification, and insufficient ADR management. Global data indicate that high-income countries have more effective pharmacovigilance systems and higher ADR reporting rates compared to low-income countries [17].

Study limitations

This study’s retrospective design relied on existing records, which may limit data completeness and accuracy. Ethical constraints prevented rechallenge for causality confirmation, posing a limitation for definitive assessment.

## Conclusions

The study provides important insights into serious and non-serious ADRs reported in 2022 at a tertiary care center in India. Adults were predominantly affected, with antibiotics, especially cephalosporins, being common culprits. Most ADRs were recognized and labeled, confirming known drug-event relationships, though a few potentially novel or rare ADRs were noted. These findings reinforce the critical role of pharmacovigilance in promoting patient safety and guiding rational drug use. Strengthening pharmacovigilance frameworks and encouraging participation by both healthcare providers and patients in ADR reporting are vital to reducing ADR-related morbidity and mortality.
